# miR-190-5p Alleviates Myocardial Ischemia-Reperfusion Injury by Targeting PHLPP1

**DOI:** 10.1155/2021/8709298

**Published:** 2021-11-25

**Authors:** Yangxue Li, Zhibo Li, Jiangen Liu, Yihang Liu, Guobin Miao

**Affiliations:** ^1^Department of Cardiology, The Second Hospital of Jilin University, Changchun, China; ^2^Department of Cardiology, Beijing Tsinghua Changgung Hospital, Tsinghua University, Beijing, China

## Abstract

**Objective:**

Myocardial ischemia-reperfusion (I/R) injury (MIRI) refers to the more serious myocardial injury after blood flow recovery, which seriously affects the prognosis of patients with ischemic cardiomyopathy. This study explored the new targets for MIRI treatment by investigating the effects of miR-190-5p and its downstream target on the structure and function of myocardial cells.

**Methods:**

We injected agomir miR-190-5p into the tail vein of rats to increase the expression of miR-190-5p in rat myocardial cells and made an I/R rat model by coronary artery occlusion. We used 2,3,5-triphenyl tetrazolium chloride staining, lactate dehydrogenase (LDH) detection, echocardiography, and hematoxylin-eosin (HE) staining to determine the degree of myocardial injury in I/R rats. In addition, we detected the expression of inflammatory factors and apoptosis-related molecules in rat serum and myocardial tissue to determine the level of inflammation and apoptosis in rat myocardium. Finally, we determined the downstream target of miR-190-5p by Targetscan system and dual luciferase reporter assay.

**Results:**

The expression of miR-190-5p in an I/R rat myocardium was significantly lower than that in normal rats. After treatment of I/R rats with agomir miR-190-5p, the ischemic area of rat myocardium and the concentration of LDH decreased. The results of echocardiography and HE staining also found that overexpression of miR-190-5p improved the structure and function of rat myocardium. miR-190-5p was also found to improve the viability of H9c2 cells *in vitro* and reduce the level of apoptosis of H9c2 cells. The results of Targetscan system and dual luciferase reporter assay found that miR-190-5p targeted to inhibit pleckstrin homology domain leucine-rich repeat protein phosphatase 1 (PHLPP1). In addition, inhibition of PHLPP1 was found to improve the viability of H9c2 cells.

**Conclusion:**

Therefore, miR-190-5p can reduce the inflammation and apoptosis of myocardium by targeting PHLPP1, thereby alleviating MIRI.

## 1. Introduction

Myocardial ischemia-reperfusion (I/R) injury (MIRI) means that after acute coronary artery occlusion, reperfusion leads to more serious myocardial injury than ischemia itself, including ischemia-induced injury and reperfusion injury [1]. Reperfusion of blood flow can cause damage to uninvolved myocardial cells and can also aggravate the damage of involved myocardial cells [2]. In addition, some cytokines affect nonfocal organs with blood flow, causing organ damage. Reperfusion injury can be up to 50% of the total myocardial injury, often causing serious adverse outcomes such as heart failure, arrhythmia, or even circulatory arrest [3].

Pleckstrin homology domain leucine-rich repeat protein phosphatase (PHLPP) was first discovered by Gao et al. in 2005 and is a novel intracellular serine/threonine phosphatase [4]. PHLPP1 promotes apoptosis induced by mTOR2 by interfering with the downstream signal of phosphatidylinositol-3-kinase/protein-serine-threonine kinase (PI3K/Akt) signaling pathway and rat sarcoma protein/extracellular regulatory protein kinase pathway, thereby playing a role in inhibiting tumor growth [5]. In addition to the initial inhibitory effect on tumor growth, PHLPP1 has been also found to have a close relationship with myocardial injury in recent years. A study found that luteolin can alleviate doxorubicin-induced myocardial injury by regulating PHLPP1/Akt signaling pathway [6]. Zhang et al. found that the inhibition of PHLPP1 can alleviate myocardial dysfunction [7]. It was also revealed that PHLPP1 ablation can prevent pathological hypertrophy by promoting angiogenesis through the activation of Akt [8]. In addition, Tang et al. found that the upregulation of PHLPP1 is a key mechanism for tumor necrosis factor- (TNF-) *α* to participate in the course of MIRI [9]. Therefore, PHLPP1 plays an important role in heart disease.

MicroRNA (miRNA) is a class of small noncoding single-stranded RNA molecules that are sheared from precursor single-stranded RNA molecules, negatively regulating gene expression and regulating the translation and stability of specific genes [10]. A study has shown that miRNA can specifically inhibit or target the specific mRNA and participate in the regulation of cell development, proliferation, differentiation, and apoptosis and other important cell signaling pathways [11]. In recent years, significant changes of various miRNA have been found in the myocardial tissue of MIRI, suggesting that miRNA can directly or indirectly affect MIRI. miR-190-5p has been found to be involved in the occurrence and development of various diseases and to regulate the biological processes of cells, including cell proliferation, apoptosis, and metabolism [12]. Our previous study found that miR-190-5p was expressed differently in the myocardium of normal rats and I/R rats. In order to further study the effect of miR-190-5p on MIRI, we studied the effect of miR-190-5p on the viability of myocardial cells *in vivo* and *in vitro* through transfection.

## 2. Materials and Methods

### 2.1. Animals

A total of 60 male Sprague Dawley rats (8 weeks old, 200 ± 20 g) were used in this study. The animal experiments in this study were approved by the Jilin University Ethics Committee. All rats were housed in specific pathogen-free animal rooms of Jilin University Experimental Animal Center. The temperature of the animal room is 24 ± 2°C, and the relative humidity is 40-60%. We made an I/R model by ligating the rat coronary arteries. After anesthetizing the rat with 2% sodium pentobarbital (40 mg/kg), we removed the hair from the rat's chest and disinfected it with iodophor. Then, we used a small animal ventilator (CWE SAR-830, Orange, CA, USA) to maintain the rat's breathing and observed the electrocardiogram of the rat using an electrocardiograph. During the operation, the rat was maintained under continuous anesthesia by inhalation of methoxyflurane (1.5%) on an operating table. We cut the skin and sternum of the rat's left chest. After exposing the rat heart, we gently cut the heart envelope and ligated the left anterior descending coronary artery with sutures. The elevation of the ST segment of the electrocardiogram indicated myocardial ischemia. After 30 minutes, we loosened the ligature. The decrease in the ST segment of the electrocardiogram indicated the recovery of myocardial blood flow. After 180 minutes of restoration of blood flow, we detected the rat's cardiac function and collected rat serum and myocardial tissue. The rats in the sham group were only opened the chest cavity without ligating the coronary arteries. Rats in the I/R+agomir negative control (NC) group and the I/R+agomir miR-190-5p group were injected with agomir NC and agomir miR-190-5p via the tail vein 48 hours before modeling.

### 2.2. 2,3,5-Triphenyl Tetrazolium Chloride (TTC) Staining

We put the rat heart in the -20°C refrigerator for 20 minutes. The rat heart was then cut into slices with thickness of 2 mm. We used 1% TTC staining solution (Sigma-Aldrich, St. Louis, MO, USA) to stain myocardial tissue for 15 minutes. A normal myocardium was red, and an ischemic myocardium was pale.

### 2.3. Echocardiography

After anesthetizing rats with isoflurane, we used VEVO-2100 small animal ultrasound system to detect the cardiac function of mice. We use the MS-400 high-frequency probe to detect the long-axis view of the left ventricle and the short-axis view of the left ventricle near the sternum. The left ventricular ejection fraction (EF) and left ventricular fraction shortening (FS) were recorded.

### 2.4. Hematoxylin-Eosin (HE) Staining

HE staining was used to detect the structure of rat myocardial tissue. We collected rat hearts and fixed them with 4% paraformaldehyde. Then, we made the rat heart tissue into paraffin blocks. We used a microtome to make paraffin blocks into 5 *μ*m thick paraffin sections. Before HE staining, we used xylene and gradient alcohol for dewaxing and hydration. Then, we stained the cell nucleus with hematoxylin (Beyotime, Shanghai, China) and used hydrochloric acid alcohol for differentiation. Then, we used eosin (Beyotime, Shanghai, China) to stain the cytoplasm. Finally, we dehydrated the myocardial tissue with alcohol and sealed them with neutral mounting medium.

### 2.5. Cell Culture

The rat myocardial cell line (H9c2) was used in this study. Dulbecco's modified Eagle medium (DMEM) (Gibco, Rockville, MD, USA) containing 10% fetal bovine serum (Gibco, Rockville, MD, USA) and 1% double antibody (Gibco, Rockville, MD, USA) was used to culture H9c2 cells. We induced the H9c2 cell injury model through hypoxia/reoxygenation (H/R). After the cells were seeded into the 6-well plate, we cultured the cells using serum-free DMEM and placed the cells in an incubator with 5% CO_2_, 1% O_2_, and 94% N_2_. After 6 hours, we changed the medium to normal DMEM and placed the cells in an incubator with 5% CO_2_, 21% O_2_, and 74% N_2_. We used lipofectamine 3000 to transfect NC mimics, miR-190-5p mimics, NC inhibitor, miR-190-5p inhibitor, siRNA-NC, or siRNA-miR-190-5p into H9c2 cells according to the manufacturer's instructions.

### 2.6. RNA Isolation and Quantitative Real-Time Reverse Transcription-Polymerase Chain Reaction (RT-PCR)

We used the TRIzol (Invitrogen, Carlsbad, CA, USA) method to extract total RNA from myocardial tissue and H9c2 cells and dissolved the RNA with RNase-free water. Then, we used a spectrophotometer to detect the concentration of RNA. We used reverse transcriptase to reverse the mRNA to cDNA. The cDNA was stored in a -20°C refrigerator for a long time. Then, we used SYBR Green Master Mix to amplify the cDNA. We used different primers to specifically amplify the corresponding DNA fragments. The primer sequences are shown in Table 1. 2^-*ΔΔ*CT^ was used to represent the relative expression of mRNA.

### 2.7. Cell Counting Kit-8 (CCK8) Assay

H9c2 cells were seeded on 96-well plates. After treating the cells according to the experimental requirements, we added 10 *μ*l of CCK8 reagent (Dojindo Molecular Technologies, Kumamoto, Japan) to each well. After incubating the 96-well plate in an incubator for 2 hours, we measured the absorbance (OD) of each well of the 96-well plate at 450 nm using a microplate reader in the dark. There was only the medium without cells in the blank wells, while the control wells had cells without treatment: cell viability = (OD sample − OD blank)/(OD control − OD blank).

### 2.8. Enzyme-Linked Immunosorbent Assay (ELISA)

ELISA was used to detect the levels of inflammatory factors (interleukin- (IL-) 1*β*, TNF-*α*, IL-6, and IL-8) and LDH in rat serum. The standards in the ELISA kits (R&D Systems, Emeryville, CA, USA) were diluted to different concentrations, and standards were used to make a standard curve. We then determined the LDH concentration of the inflammatory factor based on the absorbance of the sample and the standard curve. The same method was also used to detect the concentration of LDH in H9c2 cell medium.

### 2.9. Terminal Deoxynucleotidyl Transferase-Mediated dUTP-Biotin Nick End Labeling (TUNEL) Assay

H9c2 cells were seeded on 24-well plates and treated according to experimental requirements. Then, we used 4% paraformaldehyde and 0.1% TritonX-100 to sequentially treat H9c2 cells. We used the TUNEL kit (Sigma-Aldrich, St. Louis, MO, USA) for staining according to the manufacturer's instructions. Apoptotic cells were positive, and the number of positive cells was proportional to the level of apoptosis.

### 2.10. Dual Luciferase Reporter Assay

We used the Targetscan system (http://www.targetscan.org/vert_72/) to predict the potential targets of miR-190-5p. PHLPP1 was found to be a target downstream of miR-190-5p. Therefore, we used the HEK 293T cell line to detect the interaction between miR-190-5p and PHLPP1. We constructed wild type (WT) mRNA 3′-UTR and mutation (MUT) mRNA 3′-UTR. Then, we used the dual luciferase reporter assay kit (R&D Systems, Emeryville, CA, USA) to detect the fluorescence intensity of WT and MUT combined with miR-190-5p according to the manufacturer's instructions.

### 2.11. Statistical Analysis

Statistical Product and Service Solutions (SPSS) 21.0 statistical software was used to analyze the results of this study. We used a *t*-test to analyze the difference between the two groups of data and used one-way analysis of variance to analyze the difference between multiple groups. *P* < 0.05 was considered statistically significant. All experiments were repeated three times.

## 3. Results

### 3.1. miR-190-5p Expression Was Reduced in I/R Rats

**Figure 1:**
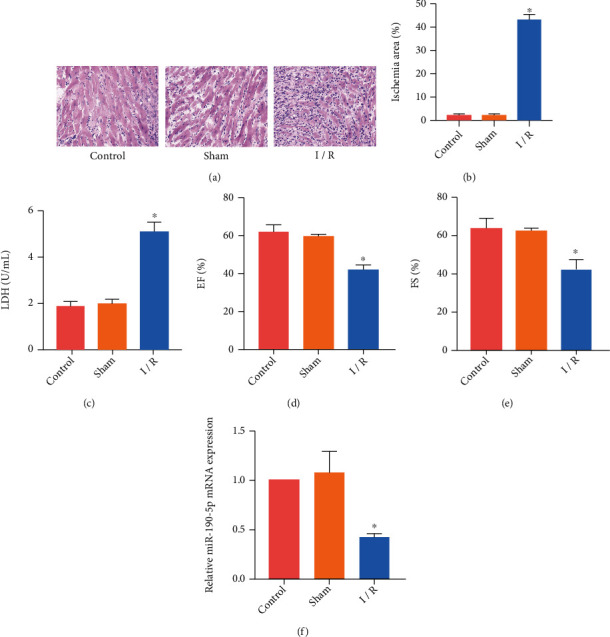
miR-190-5p expression was reduced in I/R rats. (a) HE staining of rat myocardium (200×); (b) Myocardial ischemic area of rats; (c) LDH level in rat serum; (d, e). EF and FS of rats; (f) miR-190-5p expression in a rat myocardium. (“∗” means *P* < 0.05 vs. the sham group).

We made a rat I/R model by coronary artery occlusion. HE staining detected the structure of rat myocardial tissue (Figure 1(a)). The structure of the myocardial tissue of the rats in the control group and the sham group was intact, while the myocardial cells of the I/R group were disorderly arranged and there were inflammatory cell infiltrations in the intercellular substance. TTC staining detected the ischemic area (Figure 1(b)). The ischemic area of rats in I/R group was significantly larger than that in the control group and sham group. The results of the LDH detection also showed that the serum of the I/R group of rats contained a large amount of LDH (Figure 1(c)). The results of rat cardiac function detection showed that EF (Figure 1(d)) and FS (Figure 1(e)) of I/R rats were significantly reduced. These results indicated that the I/R model was successfully made in rats. We detected the expression of miR-190-5p in rats by RT-PCR. The expression of miR-190-5p in myocardial tissue of I/R rats was significantly lower than that in the control group and sham group (Figure 1(f)).

### 3.2. Overexpression of miR-190-5p Alleviated Myocardial Injury in I/R Rats

**Figure 2:**
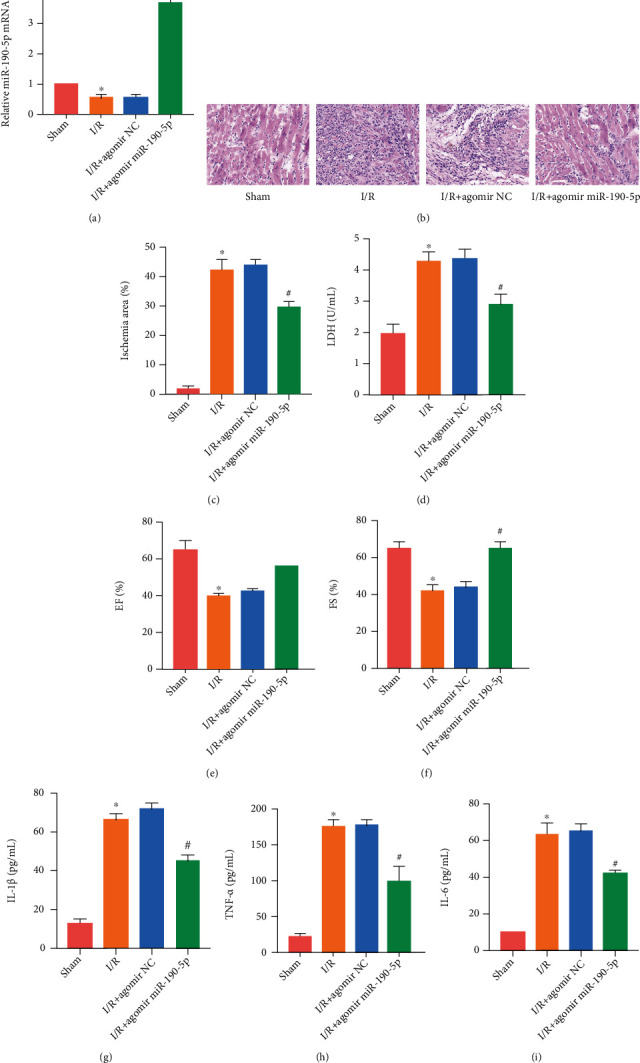
Overexpression of miR-190-5p alleviated myocardial injury in I/R rats. (a) miR-190-5p expression in rat myocardium; (b) HE staining of rat myocardium (200×); (c) Myocardial ischemic area of rats; (d) LDH level in rat serum; (e, f) EF and FS of rats; (g–i) ELISA results of IL-1*β*, TNF-*α*, and IL-6 in rat serum. (“∗” means *P* < 0.05 vs. the sham group; “#” means *P* < 0.05 vs. the I/R+agomir NC group).

We increased the expression of miR-190-5p in rats by agomir NC and agomir miR-190-5p transfection. We identified the overexpression of miR-190-5p in rat myocardial tissue by RT-PCR (Figure 2(a)). HE staining detected changes in a rat myocardial tissue structure (Figure 2(b)). After using agomir miR-190-5p to promote the expression of miR-190-5p in rat myocardial tissue, we found that the structure of rat myocardial tissue was significantly improved. The results of TTC staining also showed that miR-190-5p reduced the myocardial ischemic area in rats (Figure 2(c)). The results of LDH detection showed that miR-190-5p can reduce the level of LDH in rat serum (Figure 2(d)). Cardiac function detection results showed that miR-190-5p increased EF (Figure 2(e)) and FS (Figure 2(f)) in rats. ELISA detected the expression of inflammatory factors (IL-1*β*, TNF-*α*, and IL-6) in rat serum. The levels of inflammation in rats treated with agomir miR-190-5p were significantly reduced (Figures 2(g)–2(i)).

### 3.3. Overexpression of miR-190-5p Alleviated H/R-Induced H9c2 Cell Injury

**Figure 3:**
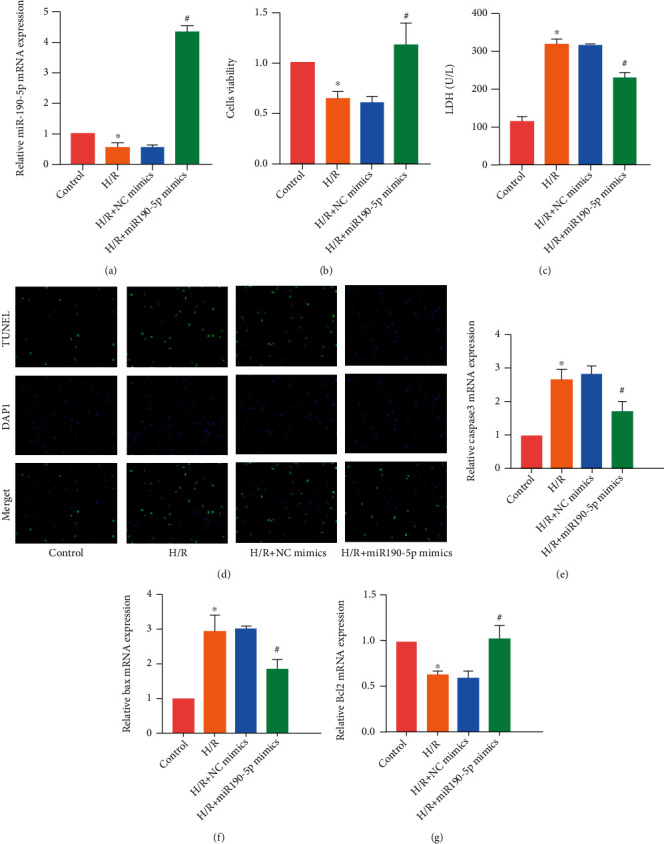
Overexpression of miR-190-5p alleviated H/R-induced H9c2 cell injury. (a) miR-190-5p expression in H9c2 cells; (b) CCK8 assay results of H9c2 cells; (c) LDH level in DMEM of H9c2; (d) TUNEL staining results in H9c2 cells (200×); (e–g) mRNA expression of caspase3, Bax, and Bcl2 in H9c2 cells. (“∗” means *P* < 0.05 vs. the control group; “#” means *P* < 0.05 vs. the I/R+NC mimics group).

To determine the effect of miR-190-5p on cardiomyocytes, we used miR-190-5p mimics to transfect H9c2 cells. RT-PCR results showed that miR-190-5p mimics significantly increased the expression of miR-190-5p in H9c2 cells (Figure 3(a)). We detected the proliferation ability of H9c2 cells by CCK8 assay. The proliferation ability of cells in the H/R group decreased significantly, and miR-190-5p mimics were found to increase the proliferation ability of H9c2 cells (Figure 3(b)). Furthermore, we detected the level of LDH in the cell medium. The LDH level in the H/R group was higher than that in the control group, while the LDH level in the H/R+miR-190-5p mimics group decreased (Figure 3(c)). TUNEL staining detected the apoptosis level of H9c2 cells. The apoptosis level of the H/R group increased significantly while miR-190-5p mimics decreased the apoptosis level of H9c2 cells (Figure 3(d)). RT-PCR detected the expression of proapoptotic molecules caspase3 and Bax, and antiapoptotic molecule Bcl2. miR-190-5p mimics were also found to reduce the mRNA expression of caspase3 and Bax and increase the mRNA expression of Bcl2 (Figures 3(e)–3(g)).

### 3.4. miR-190-5p Targets PHLPP1, and the Deficiency of PHLPP1 Alleviates H/R-Induced Cell Injury

**Figure 4:**
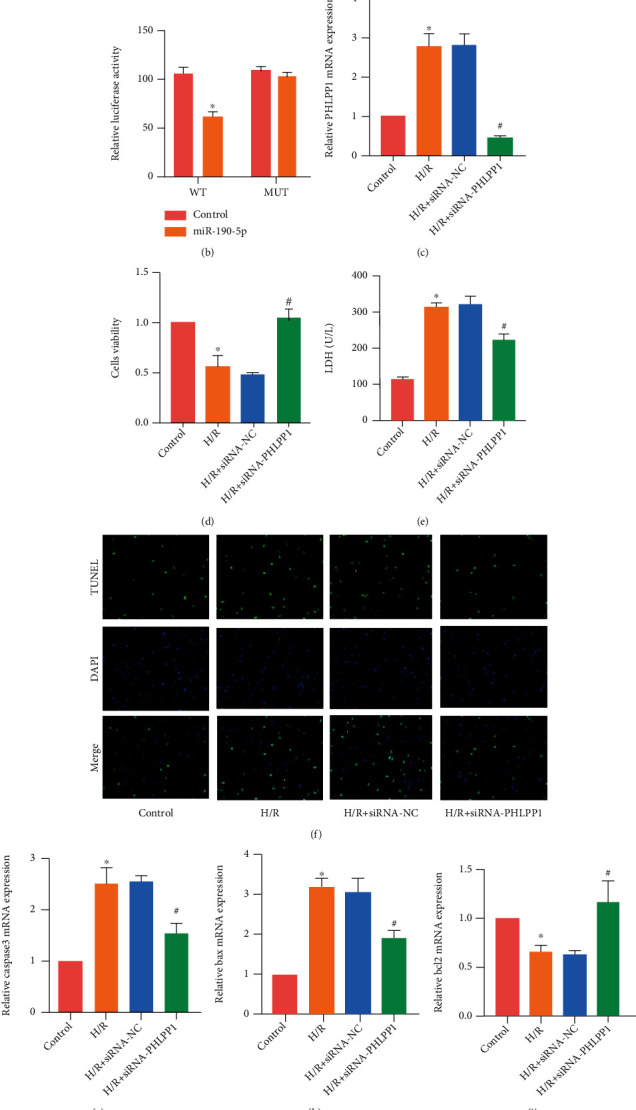
miR-190-5p targets PHLPP1 and the deficiency of PHLPP1 alleviates H/R-induced cell injury. (a) miR-190-5p has potential binding sites with PHLPP1. (b) The mRNA of PHLPP1 was degraded by miR-190-5p. (c) PHLPP1 mRNA expression in H9c2 cells. (d) CCK8 assay results of H9c2 cells. (e) LDH level in DMEM of H9c2. (f) TUNEL staining results in H9c2 cells (200×); (g–i). mRNA expression of caspase3, Bax, and Bcl2 in H9c2 cells. (“∗” means *P* < 0.05 vs. the control group; “#” means *P* < 0.05 vs. the I/R+siRNA-NC group).

We predicted the potential binding site of miR-190-5p and PHLPP1 through the Targetscan system (Figure 4(a)). Then, we clarified the effect of miR-190-5p on PHLPP1 by dual luciferase reporter assay. It was found that miR-190-5p can significantly reduce the fluorescence intensity of WT without affecting the MUT (Figure 4(b)). This indicated that miR-190-5p can degrade PHLPP1 mRNA. Then, we used siRNA-PHLPP1 to reduce the expression of PHLPP1 in H9c2 cells to clarify the effect of PHLPP1 on H9c2 (Figure 4(c)). The results of CCK8 assay indicated that the deficiency of PHLPP1 can increase the viability of H9c2 cells (Figure 4(d)). In addition, LDH detection results also found that the deficiency of PHLPP1 reduced the LDH level in H9c2 cell medium (Figure 4(e)). The result of TUNEL staining proved that the deficiency of PHLPP1 can reduce the apoptosis level of H9c2 cells (Figure 4(f)). RT-PCR results revealed that the deficiency of PHLPP1 reduced the expression of caspase3 and Bax and increased the expression of Bcl2 (Figures 4(g)–4(i)).

## 4. Discussion

MIRI is a common clinical pathological phenomenon in the cardiovascular department. It often occurs in the process of revascularization of acute myocardial infarction. It is also seen in blood flow blockage, organ transplantation, and shock treatment during surgery [13]. MIRI involves calcium overload, inflammation, oxidative stress, endoplasmic reticulum stress, mitochondrial dysfunction, and protease activation [14]. miRNA has been found to be involved in the course of MIRI in recent years and affect various aspects of myocardial injury. A study has found that miR-24-3p can target RIPK1 to inhibit MIRI in mice. miR-24-3p was found to be lowly expressed in myocardial tissues of I/R rats and overexpression of miR-24-3p protected myocardial cells from I/R injury [15]. Another study also found that miR-374 can protect myocardial cells by inhibiting SP1 and activating PI3K/Akt signaling pathway [16]. Our study focused on miR-190-5p. miR-190 has been extensively studied in many diseases, such as Parkinson's disease, chronic kidney disease, and various tumor diseases. Sun et al. found that miR-190 can alleviate nerve injury and reduce nerve inflammation in Parkinson's disease model mice [17]. Rudnicki et al. [18] found that miR-190 was lowly expressed in the kidney tissue of patients with chronic kidney disease. In our study, we made an I/R rat model by ligating coronary arteries and detected the difference in the expression of miR-190-5p in a rat myocardium. After ligating the rat coronary arteries, the expression of miR-190-5p in rat myocardial tissue was significantly reduced, suggesting that miR-190-5p was involved in the process of myocardial injury and the decreased expression of miR-190-5p may be one of the factors of MIRI. After injection of agomir miR-190-5p through the tail vein, the myocardial structure and cardiac function of the rats were significantly improved. Moreover, the level of inflammation in rats also decreased with the treatment of agomir miR-190-5p. This suggested that the increase of miR-190-5p alleviated myocardial injury in rats. In addition, in order to clarify the specific effect of miR-190-5p on myocardial cells, we used miR-190-5p mimics to treat H9c2 cells and found that the increase of miR-190-5p improved the proliferation ability of H9c2 cells and reduced its apoptosis level, which suggested that miR-190-5p can regulate the damage process of myocardial cells. The results of the Targetscan system and dual luciferase reporter assay indicated that miR-190-5p can target to inhibit PHLPP1. This may be an important mechanism by which miR-190-5p exerted myocardial protection.

The PHLPP gene is located on chromosomes 18 and 16 of the human body. The PHLPP proteins (PHLPP1 and PHLPP2) are protein phosphatases. Their physiological role is to specifically dephosphorylate phosphorylated Akt and inactivate the protein kinase activity, thereby inhibiting the growth promoting effect of Akt [5]. Brognard and Newton [19] found that when PHLPP1 is not expressed, the phosphorylation level of Akt in cells can be increased by 30-fold under the induction of agonists. A study has shown that inhibition of PHLPP1 can improve diabetic cardiomyopathy by activating PI3K/Akt signaling pathway [7]. In addition, a study has found that the deficiency of PHLPP1 can promote the proliferation of chondrocytes by increasing the expression of fibroblast growth factor 18 [20]. Therefore, we used H9c2 cells to study the effect of PHLPP1 on the viability of myocardial cells. We used siRNA to reduce the expression of PHLPP1 in H9c2 cells. The results of experiments such as CCK8 and TUNEL showed that the deficiency of PHLPP1 can increase the proliferation ability of H9c2 cells and reduce apoptosis, thus alleviating H/R-induced cell injury. Therefore, the degree of myocardial cell injury is positively correlated with the expression of PHLPP1, and targeted inhibition of PHLPP1 may be one of the methods to alleviate myocardial injury.

During myocardial I/R, aseptic inflammation is generated, which activates the innate immune response and adaptive immune response, leading to the production of a large number of inflammatory cells, as well as inflammatory reactions in other organs [21]. Excessive inflammatory stimulation leads to the activation of apoptotic cascade pathway in myocardial cells and promotes the expression and activation of caspase3. The activated caspase3 hydrolyzes various intracellular proteins and causes apoptosis. In addition, the ratio of the expression levels of Bax and Bcl2 in myocardial cells also increases with the increase of inflammation level, thereby promoting the apoptosis of myocardial cells [22]. After overexpressing miR-190-5p in rat myocardial cells or knocking down PHLPP1 in H9c2 cells, we found that the expression of caspase3 and Bax in myocardial cells decreased, while the expression of Bcl2 increased. Results of TUNEL staining also showed that the overexpression of miR-190-5p and the knockdown of PHLPP1 can inhibit the apoptosis of myocardial cells. Therefore, miR-190-5p targeted and inhibited PHLPP1 and alleviated the level of inflammation and apoptosis in myocardial tissue, which may provide a new target for clinical MIRI treatment.

## 5. Conclusions

This is the first study to investigate the miR-190-5p targeting and inhibiting PHLPP1 to regulate MIRI. miR-190-5p reduced the expression of inflammatory factors in a rat myocardium and serum by targeting PHLPP1, thereby reducing the apoptosis of rat myocardial cells. Therefore, miR-190-5p can improve the structure and function of myocardial tissue and may become a potential therapeutic target of MIRI.

## Figures and Tables

**Table 1:** Primer sequence.

Name	Sense/antisense (S/AS)	Sequences (5′-3′)
miR-190-5p	S	GGTCTTTGATGATGATTCTGG
	AS	CTAGGCACAGTATTGAAGGTT

PHLPP1	S	GGCCAAGGAGAAGGAGAGA
	AS	TGGTCCCCACAGCAGAA

Caspase3	S	TGGACAACAACGAAACCTC
	AS	ACACAAGCCCATTTCAGG

Bax	S	CGGCTGCTTGTCTGGAT
	AS	TGGTGAGTGAGGCAGTGAG
Bcl2	S	GTCACAGAGGGGCTACGA
	AS	GTCCGGTTGCTCTCAGG

GAPDH	S	GTTGTGGCTCTGACATGCT
	AS	CCCAGGATGCCCTTTAGT

U6	S	GCTTCGGCAGCACATATACTAAAAT
	AS	CGCTTCACGAATTTGCGTGTCAT

## Data Availability

The datasets used and analyzed during the current study are available from the corresponding author on reasonable request.
